# SAGE detects microRNA precursors

**DOI:** 10.1186/1471-2164-7-285

**Published:** 2006-11-07

**Authors:** Xijin Ge, Qingfa Wu, San Ming Wang

**Affiliations:** 1 Center for Functional Genomics, Division of Medical Genetics, Department of Medicine, ENH Research Institute, 1001 University Place, Evanston, IL 60201 USA; 2 Robert H. Lurie Comprehensive Cancer Center, Northwestern University Feinberg School of Medicine, 1001 University Place, Evanston, IL 60201 USA

## Abstract

**Background:**

MicroRNAs (miRNAs) have been shown to play important roles in regulating gene expression. Since miRNAs are often evolutionarily conserved and their precursors can be folded into stem-loop hairpins, many miRNAs have been predicted. Yet experimental confirmation is difficult since miRNA expression is often specific to particular tissues and developmental stages.

**Results:**

Analysis of 29 human and 230 mouse longSAGE libraries revealed the expression of 22 known and 10 predicted mammalian miRNAs. Most were detected in embryonic tissues. Four SAGE tags detected in human embryonic stem cells specifically match a cluster of four human miRNAs (mir-302a, b, c&d) known to be expressed in embryonic stem cells. LongSAGE data also suggest the existence of a mouse homolog of human and rat mir-493.

**Conclusion:**

The observation that some orphan longSAGE tags uniquely match miRNA precursors provides information about the expression of some known and predicted miRNAs.

## Background

MicroRNAs (miRNAs) are endogenous, ~22 nucleotide (nt) noncoding RNAs that play important roles in gene expression regulation by base-pairing with messenger RNAs [1]. A single miRNA can down-regulate a large number of target mRNAs [2]. Since most miRNA precursors can be mapped to ~60–120 nt long conserved genomic regions and can be folded into hairpin structures, miRNAs can be predicted from genomic sequences with high sensitivity [3-9]. Experimental confirmation and functional analysis of these predicted miRNAs, however, remains a challenge.

Serial analysis of gene expression (SAGE) collects short 14–21 nt tags from 3' ends of transcripts after certain restriction enzyme cutting sites; the most frequently used site is "CATG" which is recognized by NalIII [10] recently developed variation of this technique known as longSAGE collects 21 bp tags, which are long enough for genomic mapping and specific annotation [11]. Unlike DNA microarray that depends on a pre-defined gene set, SAGE is an exploratory method for transcriptome analysis. Many orphan SAGE tags that cannot be associated with any known transcripts represent potential novel transcripts [12].

Primary miRNAs transcribed by polymerase II are processed by the nuclear Drosha enzyme to give pre-miRNAs, which are then exported into cytoplasm and lead to mature miRNAs. At least some primary miRNAs are known to be capped and polyadenylated in the nucleus [13]. As recent analysis of EST identified 26 known miRNAs [14], SAGE might also be able to detect some primary miRNAs. To investigate whether this is the case, we mined the large number of human and mouse longSAGE tags deposited in public databases and compared these tags with the sequences of pre-miRNAs.

## Results and discussion

To identify a set of SAGE tags that could theoretically be contributed by miRNAs, we searched for "CATG" sites in known miRNA precursors. Among the 332 known human miRNAs in the miRBASE [15], 92 (28%) bear such sites. Similarly, 64 (24%) of the 270 known mouse miRNAs could contribute to SAGE tags. To increase coverage, we also included longSAGE tags uniquely mapped to genomic loci that are very close (within 30 bp) to known hairpin sequences. This is because the complex process of miRNA biogenesis is still not well understood and the complete primary transcription units, which can be significantly longer than the ~60–120 bp hairpin sequence, have not been defined for most miRNAs. After extension, the number of human and mouse miRNAs associated with longSAGE tags increased to 130 (39%) and 99 (37%), respectively. Thus, SAGE can theoretically detect about one-third of known miRNAs. Additional File 1 lists all these miRNAs and corresponding longSAGE tags.

These virtual tags were then compared with experimentally observed tags in 29 human and 120 mouse longSAGE libraries in the Gene Expression Omnibus database [16] and in 110 mouse longSAGE libraries representing various tissues in multiple developmental stages from the Mouse Atlas of Gene Expression website [17]. We identified nine longSAGE tags matched to human miRNAs and 16 matched to mouse miRNAs. These tags were then mapped to human or mouse genomic sequences and annotated with available mRNAs and ESTs. After removing tags that may have originated from known genes (e.g., mapping to the sense strand of an exon including UTR) and those that mapped to multiple genomic loci, we identified eight human and 14 mouse longSAGE tags that represent known miRNAs (Table 1).

**Figure 1:**
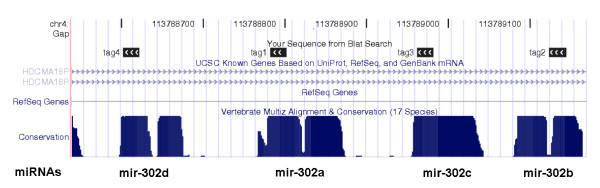
Four human longSAGE tags specifically mapped to a cluster of four miRNAs on Chromosome 4. These evolutionarily conserved miRNAs are transcribed from the antisense strand of an intron of HDCMA18P gene.

Among the eight human miRNAs whose expression was detected by SAGE tags, four (mir-302a, b, c&d) mapped to a 600 bp region of Chr. 4q25 (Fig. 1). Another member of the cluster, mir-367, was not detected because of the lack of the "CATG" site. This miRNA cluster is known to be specifically expressed in human embryonic stem cells [18], which is in accord with the source of the SAGE libraries in which the tags were observed (see Table 1, detailed information about SAGE libraries is available in Additional File 2).

The large amount of mouse longSAGE data provides rich information about the particular tissue and developmental stage of the expression of 14 known miRNAs. In the mouse embryo at Theiler Stage 14, for example, we observed the expression of mir-133a-2 and mir-351 in heart ventricle. At the same stage, SAGE detects the expression of mir-29b-2 in heart bulbous cordis. The expression of mir-29b and mir-133 in the heart has been confirmed by northern blot [19].

LongSAGE data also indicate the expression of "known" but unconfirmed miRNAs, such as the expression of let-7i in human embryonic stem cells and fetal brain tissues. Although listed as known miRNAs in the miRBASE [15] based on the mouse homolog, its expression has not yet been experimentally confirmed in humans. Similarly, longSAGE tags also suggest the expression of two human (mir-7-1 and mir-125a) and three mouse (mir-331, mir-351, and mir-495) miRNAs that have not been experimentally confirmed (Table 1). LongSAGE data thus provide hints about the expression of unconfirmed miRNAs.

LongSAGE data also provide evidence for the existence of some predicted miRNAs. Two human and seven mouse miRNAs predicted by Lim et al. [4], Berezikov et al. [7] and Sewer et al. [9] are supported by SAGE tags (Table 1). One mouse miRNA candidate, cand202-MM, predicted by both Berezikov et al. [7] and Sewer et al. [9], is highly homologous to human and rat mir-493. The presence of such a SAGE tag in two mouse SAGE libraries strongly supports the existence of mouse mir-493. Two mouse SAGE tags map to genomic loci that are highly homologous to predicted human (cand847-HS) and rat (cand913-RN) miRNAs. The information about the tissue and stage of expression might facilitate the experimental confirmation of these predicted miRNAs.

The use of SAGE tags to detect miRNA precursors is limited, however. For example, longSAGE tags are subject to sequencing errors. Also, 21 bp tags do not provide full sequences of miRNA precursors. Therefore, further studies are needed to confirm our findings.

## Conclusion

In summary, the available longSAGE tags indicate the expression of eight human and 14 mouse known miRNA precursors and provide evidence for the existence of two human and seven mouse predicted miRNAs. Although limited in the number of miRNAs, SAGE data provide useful information on the expression of miRNA. Together with recent longSAGE-based studies that identifies many novel antisense transcripts in mouse [21] and human [22], this study again shows that longSAGE is an effective technology for exploratory transcriptome analysis.

## Methods

Genomic coordinates of 332 human and 270 mouse hairpin sequences were downloaded from the miRBase (Ref. 15) as our collection of known miRNAs. Because pre-miRNAs could be longer than these hairpin sequences, these sequences were extended by 30 bp in both directions on corresponding genomic sequences. In addition, miRNAs predicted by Lim et al. [4], Berezikov et al. [7] and Sewer et al. [9] were downloaded from the respective journal web sites. These sequences were then searched for the "CATG" site and 17 bp tags after each of these sites was extracted. Such virtual SAGE tags are linked to miRNAs for further analysis.

The 29 human and 120 mouse longSAGE libraries were retrieved from the gene expression omnibus database (Ref. 16). Another 110 mouse longSAGE libraries were downloaded from the Mouse Atlas of Gene Expression web site (Ref. 17). Pooling multiple libraries for each species led to a total of 632,813 unique human tags and 1,902,036 unique mouse tags. These experimental tags were then compared to the virtual tags extracted from miRNA sequences. Only virtual tags whose sequence is identical to the sequence of real tags were considered confirmed.

For annotation, matched human and mouse tags were mapped to human (Mar. 2006 assembly, hg18) and mouse (Aug. 2005 assembly, mm7) genomic sequences, respectively, using BLAT [20]. All tags mapped to multiple genomic loci or exons of known genes were excluded. Tags mapped to UTR regions were retained only if the tag was transcribed from the opposite strand.

## Authors' contributions

XG, QW and SMW conceived the study and participated in study design. XG did computational analyses. XG and SMW wrote the manuscript. All authors read and approved the final manuscript.

## Supplementary Material

Additional File 1 Virtual SAGE tags extracted from known miRNA precursors. This file contains 310 longSAGE tags extracted from known miRNA precursor sequences. Click here for file

Additional File 2 Detailed description of SAGE libraries that includes tags representing miRNAs precursors. This file gives detailed information on the type of tissue and stage of development (if available) that the SAGE tags listed in Table 1 are detected. SAGE library IDs are also given for further enquiry to the original databases. Click here for file

## Figures and Tables

**Table 1:** LongSAGE tags matched to known and predicted miRNA precursors.

miRNA ^(1)^	longSAGE tags ^(2)^	Chr.	EST	#libs	Tag counts	Tissue (mouse Theiler Stage)
**Human SAGE tags matched to known miRNAs**						
hsa-mir-302a	TTTTGGTGATGGTAAGT	4q25	No	1	1	Embryonic stem cell
hsa-mir-302b ^(3)^	GAAGTGCTTTCTGTGAC	4q25	Yes	5	9	Embryonic stem cell
hsa-mir-302c	TTTCAGTGGAGGTGTCT	4q25	Yes	1	2	Embryonic stem cell
hsa-mir-302d	TTTGAGTGTGGTGGTTC	4q25	No	4	6	Embryonic stem cell
hsa-mir-7-1 ^(4)^	CCTCTACAGGACAAATG	9q21	No	3	3	White blood cell, breast tumor, stem cell
hsa-let-7i ^(4)^	GCCCTGGCTGAGGTAGT	12q14	No	4	4	Embryonic stem cells and Fetal brain
hsa-mir-21	GCTGTACCACCTTGTCG	17q23	Yes	2	2	White blood cell, breast tumor
hsa-mir-125a ^(4)^	TTGCCAGTCTCTAGGTC	19q13	No	1	1	breast tumor (myofibroblast)
**Human SAGE tags matched to predicted miRNAs**						
Lim et al. [4]	CTACTCTCACTGAGTAC	5p21	No	1		Embryonic stem cell
cand525-HS	CGGAGCCCCCGGGCTTG	11q13	No	4		Embryonic stem cell and breast & lung cancer
**Mouse SAGE tags matched to known miRNAs**						
mmu-mir-29b-2 ^(3)^	GTGGCTTAGATTTTTCC	1qH6	Yes	2	2	Heart bulbous cordis (TS14 embryo)
mmu-mir-205	GAGCTGCCAGCGGTGGA	1qH6	Yes	7	17	Brain, forelimb & skin (embryo)
mmu-mir-130a	CCTTTGCTGCTGGCCGG	2qD	Yes	1	1	Branchial Arch embryonic tissue
mmu-mir-133a-2	GCCCAGCCAGAGGACAC	2qH4	Yes	4	4	Heart ventricle (TS14-19), Sk. muscle (TS25)
mmu-mir-29a	ACCTCTTGTGACCCCTT	6qA3	No	1	1	Ovary (21 days post natal)
mmu-mir-425	GAAAGTGCTTTGGAATG	9qF2	Yes	3	8	Visual cortex(27days post natal), Pancreas (TS20)
mmu-mir-331^(4)^	CAAGCTGAAAGCACTCC	10qC2	Yes	1	1	Brain – Amygdala (Post natal day 7)
mmu-let-7i	GCCCTGGCTGAGGTAGT	10qD2	Yes	4	4	Sm. Intestine (TS24), Lung (TS26), Neural tube(TS13), Pancreas
mmu-mir-21	GCTGTACCACCTTGTCG	11qC	Yes	2	2	Placenta (TS22), large intestine (TS24)
mmu-mir-196a-1	GCAGTTACTGCTTCTTG	11qD	No	3	3	Endoderm (Definitive), kidney (TS24), testis
mmu-mir-337 ^(3)^	CAGGAGTTGATTGCACA	12qF1	No	1	1	Adrenal gland (TS22 embryo)
mmu-mir-485 ^(4)^	TGTGATACTTGGAGAGA	12qF1	No	1	1	Skin (TS21)
mmu-mir-351 ^(3,4)^	GCACCTCCGTTTCCCTG	XqA5	Yes	2	2	Heart ventricle (TS14 embryo)
mmu-mir-92-2	CCCATTCATCCACAGGT	XqA5	No	1	1	Thymus (TS23 embryo)
**Mouse SAGE tags matched to predicted miRNAs**						
cand185-MM	TGACTGCCTGTCTGTGC	1qH2	Yes	1	1	Fibroblast cell line (embryonic)
cand847-HS	GTGAGCAGATGATGCAT	2qH1	Yes	1	1	Brain – Whole (TS20 embryo)
cand136-MM	AACATTATTTCTTGTGT	4qD2	No	1	1	Adult testis
cand407-MM	GAGTCTTCCAAGCCAAG	4qF4	No	3	3	Adult bladder & mammary gland
cand913-RN	TGACGTCTGAGGAGCGG	11qE2	Yes	1	1	Brain telencephalon dorsal (TS20 embryo)
cand219-MM	GCCAATCTCCTTTCGGC	12qF1	Yes	2	6	Visual cortex (27 days post natal)
cand202-MM	GTAGGCTTTCATTCATT	12qF1	No	2	2	Branchial arch embryonic tissue (TS15)
cand239-MM	CAGCTTTGGAGACGCCA	16qC3	No	1	1	Brain – Preplate (TS21 embryo)
cand525-MM	CGGAGCCCCCGGGCTTG	19qA	No	9	9	Multiple normal tissues

^(1) ^Except the one marked as Lim et al. [4], all predicted miRNAs are based on Berezikov et al. [7]. Cand219-MM and Cand202-MM are also predicted by Sewer et al. [9].

^(2) ^"CATG" sites before each SAGE tags had been omitted.

^(3) ^SAGE tags match miRNA hairpin sequences without extension.

^(4) ^These miRNAs are listed as known in miRBASE based on their homolog to entries in other species.
